# Correction: Unveiling the obstacles encountered by women doctors in the Pakistani healthcare system: A qualitative investigation

**DOI:** 10.1371/journal.pone.0318366

**Published:** 2025-01-24

**Authors:** Ali Raza, Junaimah Jauhar, Noor Fareen Abdul Rahim, Ubedullah Memon, Sheema Matloob

There is an error in affiliation 3 for author Noor Fareen Abdul Rahim. The correct affiliation 3 is: Graduate School of Business, Universiti Sains Malaysia, Penang, Malaysia.

## References

[1] Raza A, Jauhar J, Abdul Rahim NF, Memon U, Matloob S (2023) Unveiling the obstacles encountered by women doctors in the Pakistani healthcare system: A qualitative investigation. PLOS ONE 18(10): e0288527. doi: 10.1371/journal.pone.0288527 37796908 PMC10553294

