# Supplementary material for: Unveiling the obstacles encountered by women doctors in the Pakistani healthcare system: A qualitative investigation
Source: PLoS One. 2023 Oct 5;18(10):e0288527. doi: 10.1371/journal.pone.0288527 (PMC10553294; doi:10.1371/journal.pone.0288527)
Supplement: Attachment — Submitted filename: Response to Reviewers.docx [file pone.0288527.s001.docx]

**Editor and Reviewers Comments and Response**

Dear Editor and Reviewers,

Please accept our heartfelt appreciation for the detail and constructive feedback on the manuscript. We are sure that your insightful remarks have immensely improved the quality of our work and we are truly grateful for the time and effort you dedicated to the review process.

While going through the review process, we came to the realization that there was a problem with the language used and the explanation of some terminologies. However, upon carefully examining the comments, we discovered that that there were some issues which we rectified and also found that some of the concerns were already addressed but did not communicate it well. However, we are open to any further suggestions from your side.

Please review the table containing comments and corresponding responses.

| **S.No:** | **Feedback** | **Responses** |
| --- | --- | --- |
| 1. | The abstract states that 59 interviews where done, while 69 participants were mentioned in the method section. Rather state that 69 potential participants were recruited while 59 interviews were done utilized for this study. People that did not finally grant the interview for the study are not referred to as participants. | Thank you for the identification. We have now corrected the discrepancies in whole document. Certainly, there were issues regarding the women doctors approached/ contacted, screened out, declined, and interviewed. Infact, the pilot interviews were mentioned but were not included in the study. These inconsistencies were also thoroughly checked and rectified to our best.  For the clarity we have inserted a new table (Table 5) under the new heading of participants for more simplicity. |
| 2. | If in-depth interviews were really done, then interview guide (NOT PROTOCOL) should be the instrument used for data collection. The interview guide allows for free flow of information from the participant without following any protocol so that even relevant information mentioned by the interviewee, which the interviewer might not have anticipated before the interview can still be explored deeply. The interview guide will then be just a guide to enable in-depth interview. | Dear Editor, thank you for pointing out. In real sense, it was interview guide because as you mentioned there was free flow of the information for example, each interviews provides us insights to update our interview guide and based on the information we were added/modified some of the questions. These modifications helped us to explore the phenomena more deeply and we felt were closer to participants views. (New text added in heading 2.4)  However, we used the word protocol as we mean as overall conduct or process of interview. i.e., *How the interviews were carried out from approaching participants, conducting, and closing.*  Based on your indications, we have made changes in the texts. |
| 3. | In line 186, you referred to the instrument for data collection as "question guide." while it was referred to as 'interview protocol" in other places.  Consistency and following the suggestion in point number 2. | Thank you for the indication. We have now corrected the discrepancies. |
| 4 | Consider removing table 2. It will be perfect to be in your PhD thesis. The mention of it in line 186 is sufficient for an article. | Thank you for the suggestion. The said table is now removed. |
| 5. | Please first table was numbered "2.1" Please number the tables properly. Let it be tables 1, 2, 3 etc. | Thank you for the suggestion, we have now updated the table and figures numbers accordingly. |
| 6. | Table 2.2 referred to the instrument for data collection as "interview topics." Please did you do an semi-structured in-depth interview? Where are the probes that were used to illicit more information during the interview? Open ended leading questions with potential probes should be stated in the interview guide for semi-structured interviews. | Dear Editor, there seems to have been a slight miscommunication. The themes or domains mentioned earlier are not the data collection instrument itself, but rather the focus areas of our interview guide. The actual instrument used for data collection consisted of semi-structured in-depth face-to-face interviews. The term "topics" was used to refer to the main categories, under which several questions and potential probes were included for further inquiry. Due to the length constraints of the article, only the categories were mentioned.  But now, to provide more clarity, we have now updated Table 2 to include some of the specific questions and probes that were utilized during the interviews. Here, it is important to note that still we have added key questions in the brief interview guide considering the length of the article. In addition, a interview guide itself is comprehensive, has undergone expert review, and was piloted prior to the actual data collection process. |
| 7 | In line 198, the objective is not to find out why women do are out of practice, but rather why their is a shortage of women doctors... | Thank you for the indication. We have now corrected the text. |
| 8 | Line 2013, change assent to consent. Minors (those below the legal age t give consent) give assent | Thank you for the raising the important point. We have now updated the text. |
| 9 | Line 203 to 205, "Following their assent, participants were assured of their identity 204 and discussion confidentiality and informed that their conversation would be presented collectively, 205 i.e., a research report." You don't assure people of their identity. You assure them that you will maintain confidentiality, their data would be made anonymous and used only for research purposes. | Dear Editor, thanks for the important suggestions. We have corrected the texts as previously there was a problem of language and clarity. |
| 10 | 10. Line 206 " Additionally, it was clear to them that the interview would be audio-recorded for later analysis." Rewrite this sentence to say that the participants were informed that the interview would be audio-taped/recorded. | Thanks for the advice, the said sentence is now re-written as per your recommendation. |
| 11. | Did you obtain consent for the audio-recording from the participants? Was it a written consent? This is different from the consent for audio-recording. | Dear Editor,  We confirm that written consent has been obtained from the women doctors participating in the study. This step is a requirement outlined by the Ethical Review Board (ERB) and aligns with the guidelines provided Graduate School of Business (GSB), Malaysia. The consent form has undergone review and approval by the ERB. It should be noted that the consent form includes explicit information regarding audio recording and provides participants with the opportunity to indicate their preference by either ticking or unticking the option for recordings. We have now attached the consent form your reference. |
| 12. | Line 107, What do you mean by "audio records would be erased as soon as the investigation ends?" When would you say the investigation has ended? What is the policy of the Human Research Committee of your institution concerning audio-recorded data? Please when submitting the revised version, attach the consent form used for the interview and the consent form used for audiotaping. Also provide a copy of the ethical approval certificate for this study. | Dear Editor, according to the regulations set forth by the Ethics Review Board (ERB), it is required to delete the audio recordings within three months after the interviews have been transcribed. This ensures that the recordings are not utilized for any purposes other than academic ones.  We formulated this statement to uphold ethical considerations in our research and comply with the guidelines of the Ethics Review Board (ERB).  Nevertheless, the researchers maintain full ownership and control over the transcriptions as the collected data is their property. It is important to note that only one version of the consent form was used, which explicitly covers the recording of audio during the interviews. Additionally, for further clarity and reference, the ethical approval has been provided. |
| 13. | Line 219, how many participants were hesitant to have their interviews recorded? Did they consent to audio-recording before the interview began? | Dear Editor,  Three participants exhibited reluctance in physically signing the written consent form prior to the interviews. Furthermore, they did not grant permission for audio recording. Nevertheless, they expressed their willingness to provide oral consent instead. |
| 14. | Was the interview face to face? Who were present during the interview (e.g. interviewer and interviewee only etc). As stated previously in the manuscript, did the interviewer travel to all the major cities in Pakistan to collect this data? Were all the participants fluent with Urdu and English? | Dear Editor,  Yes, all interviews were conducted in person, with both the interviewer and interviewee present during the interview sessions. The data collection process posed significant challenges due to extensive travel requirements, spanning over 1000 kilometers across all four provinces of Pakistan, including Azad Kashmir, which is administered by Pakistan but considered a self-governing state. (We have now added this information to the data collection process heading).  It is noteworthy that all participants, English serves as the medium of instruction in medical degree programs in Pakistan and being doctors, exhibited a high level of proficiency in both English and Urdu languages. |
| 15. | Now in line 221, you mentioned 59 participants again | Thank you for indication, we have updated the text. |
| 16. | Add citation for that definition of theoretical sampling. I think the definition given speaks more to "data saturation" method. Theoretical sampling involves collecting data and analysing simultaneously to determine what to collect next in order to develop a theory. Please clarify and state what you did. | Dear Editor, thank you for pointing out an important concern.  Following thorough consideration and discussion, we have reached a consensus that the study indeed utilized purposive sampling technique. Although it was initially mentioned as such in the doctoral thesis, the extension of the study and the inclusion of new authors prompted us to reconsider the approach. However, we have now revised this section in the manuscript.  We opted for purposive sampling because “*researchers select the participant sampling criteria prior to conducting research”* On the other hand, *Theoretical sampling is a form of sampling in qualitative research that is not bounded by the limits of a priori selection. Rather, theoretical sampling entails jointly collecting and analyzing data to decide what data to collect next and where to find them to develop theory* (Glaser & Strauss, 1967/2012).  Prior to study, we carefully set the criteria for participants selection, it would be purposive sampling.  Reference:  Conlon C, Timonen V, Elliott-O’Dare C, O’Keeffe S, Foley G. Confused About Theoretical Sampling? Engaging Theoretical Sampling in Diverse Grounded Theory Studies. Qualitative Health Research. 2020;30(6):947-959. doi:10.1177/1049732319899139 |
| 17 | Line 229 -232, how did you decide on which cities to collect data since they all have different languages, culture, social life etc. Authors now mentioned that data was collected from ALL FOUR provinces  of Pakistan, whereas they previously stated that data was collected in MOST of the provinces in Pakistan. I would suggest you remove remove some words that might not be necessary (see line 210) and add some information about the cultural background of where the data was collected. This would help contextualize and enable the reader to have a better understanding of the study. | Dear Editor, Thank you for comment.  The decision to select the cities were based on two main reasons. First, we came to point that the collecting data from various locations of Pakistan was essential due to the extensive and country-wide existence of the problem. In addition, as all four province of Pakistan have different local languages but single national language i.e., Urdu, and official language is English. So, collecting data from all origins enriched the data especially exploring and understanding various obstructions. Therefore, it was vital to obtain the narratives from different locations to thoroughly examine the matter.  Data was collected from all provinces of Pakistan. However, Islamabad is the federal territory does not come in any province. Similarly, Azad Kashmir is again a self-governing state administrated by Pakistan but does not come in any province of Pakistan.  In addition, we selected participants who meet specific criteria by leveraging our personal connections. Additionally, snowball sampling or participant-driven sampling techniques also enabled us to identify and approach potential women doctors who could participate in our study. These approaches essentially provide guidance on the selection of interviewees and locations.  As per your recommendations, the matter in line 210 is replaced by the cultural backgrounds of each province in the data collection process heading. |
| 18 | Table 3.3. Some columns have 3 cities/provinces with 2 interviews (both categories made up the 2) done in them? Also, seperate the provinces from the cities to give a clear picture. | Dear Editor, Thank you for the advice. Yes, apart from the Sindh and Punjab province. KPK- Northern side- hilly area where there is only one developed city Peshawar (there were 2 interviews). Similarly, Baluchistan- a less-developed hilly area nonboring Afghanistan has one developed city Queeta (There was 2 interviews over there also). We have added each province brief introduction in data collection process as per your advice.  As per your recommendation, the entire table is now reconstructed. We have now deleted one column of pilot interviews because they were not included in the data analysis creates confusion. |
| 19 | The number of interviews reported in table 3.3 do not add up at all. | Dear Editor, thank you for the identifying the error. It is now rectified. |
| 20 | Line 229, the quote do not match the description given above it. Please look out for this mistake all through the result section and correct it | Dear Editor, Thank you for the identification. We have now corrected the discrepancies in result section to our best and replaced some quotations if they do not match the description and add new ones. |
| 21 |  |  |
| 22 | Lines 479 to 491 seems to speak more to socio-economic and strict parental control as barriers, instead of the hijab restriction which was the theme for that section. | Dear Editor Thank you for the comments.  The concept of hijab is multifaceted and intricate, given its numerous definitions and extensive range of applications. We came to know from the data that parents and present societal structures usually enforce women to observe hijab through different means and ways. For example, women doctors’ mobility was restricted under hijab, that involves, use of public transport and restriction on performing night duties. In addition, parental influence and surveillance to observe hijab due to religion and prevailing societal structure for women. Therefore, we put these sub-themes under the heading of hijab.  However, we will be happy to hear your viewpoint and suggestion in this regard. |
| 23 | Add quotes to support the information in theme 4.2.3. Look out for this all through the result section and fix it. The quotes are the data. | Dear Editor, thank you for the suggestion, we have now added (3) relevant quotes to this theme. |
| 24 | You can present a table to show the topics presented in the results and discussion section as themes and their subthemes. Better organization of the data will make it easier for the readers to follow through. | Dear Editor, Thank you for the suggestion. We have presented table to shows the results and discuss the findings accordingly. This table has allowed us to effectively manage and organize the details of our findings in accordance with these themes. |
| 25 | Consider removing any information that does not speak to the topics discussed in the result and discussion. Also look through the paper and remove any unnecessary repetition of information. | Dear Editor, We have tried our best to check the paper and remove un-necessary information. |
| 26 | I do not think the two figures presented are relevant for publication in this paper. Also, did you create the figures (as they speak about the government but have no reference beneath them)? | Dear Editor, yes, I have created the images in ATLAS.ti 22. The source of these pictures were the interviews. They were discussing about the long-governmental process of transfers/recruitment. These two figures are removed as per your advice. |

**Reviewers 1 Comments**

| **S.No:** | **Feedback** | **Responses** |
| --- | --- | --- |
| 1 and 2 | As it is a qualitative study, manuscript title looks like a quantitative one. It should be more sympathetic to female doctors. such as constraint and inhibitory factors or like that.  Similarly, introduction section should be more polite towards female doctors, and it should not make them culprits for shortage of doctors in the country. For example "According to PMC (formerly PMDC), the foremost cause of the medical doctor shortage is non-practicing women doctors in Pakistan." I am sure it not the only cause of shortage of doctors in Pakistan. However, whole of the introduction section gives such impression | Dear Reviewer, Thank you for the valuable comments.  After conducting a review of the introduction section, we have incorporated additional information concerning the status of women in Pakistan, gender equality, and the various inequalities prevalent within the system. Furthermore, we acknowledge your observation that the shortage of women doctors is not the sole reason for the overall scarcity. Consequently, we have revisited the relevant sections and modified the statements to ensure they present a more neutral perspective, eliminating any potential impressions that may suggest otherwise. |
| 3 | Further, I believe researchers should not come up with the exact number (178,440) on shortage of doctors. As, countries with best healthcare systems are suffering with shortage of medical staff and achieving set standards on number of doctors is almost impossible. Further, we as researchers do not have authority to come-up with the number on shortage of staff. | Dear Reviewer, Thank you for the valuable comments. As per your suggestion, the exact number is now converted into approximate figure such as “more than 178,000 doctors”. |
| 4 | Manuscript looks a bit lengthy. Introduction section can be reduced. In my view there is no need to provide BoDs in introduction as this not the area of interest in this research. Introduction section should be reduced to motivation of the study, goal, objectives, and contribution. | Dear Reviewer, Thanks for the comments. We have now reviewed the sections. The discussion on BoDs is now erased to focus more on our research problem. Similarly, contributions and motivations is now added to the section for readers to better clarity and rationality. |

**.**

**Reviewers 2 Comments**

| **S.No:** | **Feedback** | **Responses** |
| --- | --- | --- |
| 1 | Contribution needs to be clearly identified in the introduction and then in the findings. Currently, it looks like more descriptive research whereas there is a great potential in the paper to be repositioned as a theory building paper. Some hints given at the end. | Dear Reviewer, we greatly appreciate the valuable suggestions. we have incorporated the contribution into the introduction section of our paper. |
| 2 | Theoretical lens can be expanded. Although it’s a qualitative paper based on the data, but little bit addition can add value, but I can see a very long discussion at the end which can be summarized in a form of a table and supporting studies maybe added in the column next to the findings of the study and in this way. Because adding the theoretical part may lengthen the paper in case discussion is not trimmed or summarized. | Reviewer, thank you for your suggestions. We have implemented your feedback by adding the theoretical contribution at the end and trimming the discussion section to focus on the core objective of the research. We have also included a thematic table in the findings section. However, we were afraid by adding a new table presenting findings with related studies would be reptations with previous section. We felt it could a doable the findings and discussion part were combined. However, we remain open to your guidance on this matter.  Furthermore, we concur with your assessment that this paper can be regarded as theory driven. However, we perceive this work as a foundational study that primarily centers on the exploration of various factors. It is crucial to emphasize that each theme we have examined holds the potential for independent investigation using a robust theoretical framework. For instance, one could delve into the examination of gender inequality in workplaces through the lens of Acker's theory or explore the concept of Purdah/hijab with reference to Masood (2018). As a result, our future research endeavors will be dedicated to delving deeper into the underlying mechanisms to comprehensively study the phenomenon. |
| 3 | Table 3.3 review number please, there seems discrepancy. Similarly, check total number across the paper and tally | We have addressed the identified discrepancies in the entire document. Specifically, we have resolved issues related to the number of women doctors approach, contact, screened, and also who declined for the interviews. Additionally, we have rectified the omission of pilot interviews mentioned but not included in the study. These inconsistencies have been thoroughly checked and corrected to the best of our ability. To improve clarity, we have updated the table and introduced a new table (Table 4) under a revised heading of "Participants Profile" to simplify the presentation of information. |
| 4 | “I appeared in the exam in 2002 and got the offer at the end of December 2003. I did not join. 313 as I was getting married then, even though I worked in a private hospital for a year (PISB3, 314 31 to 40 years, single).” The participant is single or married above. | Thank you for your review and for bringing up the issue. I have verified the information with the original data, and the participant's marital status was married, and it is now rectified throughout the paper. |
| 5 | Findings 4.1 and 4.1.1.1 needs to be converted into themes. Resulting themes. Like 4.1.2. For example, in effective recruitment processes at the government level etc. | Dear Reviewer, Thank for the advice. We have now developed a thematic table that shows the explored themes and sub-themes and manage our finding details accordingly. |
| 6 | Explain Hijab as readers may not understand the concept and underpinning philosophy if not from the same socio-cultural and religious background. | Dear Reviewer, Thank you for your valuable suggestion. We have added new information as per your advice. |
| 7 | The data is very rich, an integrated framework at the end of the findings will summarize the paper findings | Dear Reviewer, we have followed your advice and made our best effort to develop a framework based on the findings. As suggested, we have positioned it at the end of the findings section |
| 8 | Recommendations to improve the situation needs to be added as the paper can have practical solutions for the situation. | Dear Reviewer, Thank you for the valuable suggestion. Recommendations are combined with the practical contributions in one heading. |
